# Performance of ChatGPT on USMLE: Potential for AI-assisted medical education using large language models

**DOI:** 10.1371/journal.pdig.0000198

**Published:** 2023-02-09

**Authors:** Tiffany H. Kung, Morgan Cheatham, Arielle Medenilla, Czarina Sillos, Lorie De Leon, Camille Elepaño, Maria Madriaga, Rimel Aggabao, Giezel Diaz-Candido, James Maningo, Victor Tseng

**Affiliations:** 1 AnsibleHealth, Inc Mountain View, California, United States of America; 2 Department of Anesthesiology, Massachusetts General Hospital, Harvard School of Medicine Boston, Massachusetts, United States of America; 3 Warren Alpert Medical School; Brown University Providence, Rhode Island, United States of America; 4 Department of Medical Education, UWorld, LLC Dallas, Texas, United States of America; Beth Israel Deaconess Medical Center, UNITED STATES

## Abstract

We evaluated the performance of a large language model called ChatGPT on the United States Medical Licensing Exam (USMLE), which consists of three exams: Step 1, Step 2CK, and Step 3. ChatGPT performed at or near the passing threshold for all three exams without any specialized training or reinforcement. Additionally, ChatGPT demonstrated a high level of concordance and insight in its explanations. These results suggest that large language models may have the potential to assist with medical education, and potentially, clinical decision-making.

## Author summary

Artificial intelligence (AI) systems hold great promise to improve medical care and health outcomes. As such, it is crucial to ensure that the development of clinical AI is guided by the principles of trust and explainability. Measuring AI medical knowledge in comparison to that of expert human clinicians is a critical first step in evaluating these qualities. To accomplish this, we evaluated the performance of ChatGPT, a language-based AI, on the United States Medical Licensing Exam (USMLE). The USMLE is a set of three standardized tests of expert-level knowledge, which are required for medical licensure in the United States. We found that ChatGPT performed at or near the passing threshold of 60% accuracy. Being the first to achieve this benchmark, this marks a notable milestone in AI maturation. Impressively, ChatGPT was able to achieve this result without specialized input from human trainers. Furthermore, ChatGPT displayed comprehensible reasoning and valid clinical insights, lending increased confidence to trust and explainability. Our study suggests that large language models such as ChatGPT may potentially assist human learners in a medical education setting, as a prelude to future integration into clinical decision-making.

## Introduction

Over the past decade, advances in neural networks, deep learning, and artificial intelligence (AI) have transformed the way we approach a wide range of tasks and industries ranging from manufacturing and finance to consumer products. The ability to build highly accurate classification models rapidly and regardless of input data type (e.g. images, text, audio) has enabled widespread adoption of applications such as automated tagging of objects and users in photographs [1], near-human level text translation [2], automated scanning in bank ATMs, and even the generation of image captions [3].

While these technologies have made significant impacts across many industries, applications in clinical care remain limited. The proliferation of clinical free-text fields combined with a lack of general interoperability between health IT systems contribute to a paucity of structured, machine-readable data required for the development of deep learning algorithms. Even when algorithms applicable to clinical care are developed, their quality tends to be highly variable, with many failing to generalize across settings due to limited technical, statistical, and conceptual reproducibility [4]. As a result, the overwhelming majority of successful healthcare applications currently support back-office functions ranging from payor operations, automated prior authorization processing, and management of supply chains and cybersecurity threats. With rare exceptions–even in medical imaging–there are relatively few applications of AI directly used in widespread clinical care today.

The proper development of clinical AI models [5] requires significant time, resources, and more importantly, highly domain and problem-specific training data, all of which are in short supply in the world of healthcare. One of the key developments that enabled image-based AI in clinical imaging has been the ability of large general domain models to perform as well as, or even outperform, domain-specific models. This development has catalyzed significant AI activity in medical imaging, where otherwise it would be challenging to obtain sufficient annotated clinical images. Indeed today, Inception-V3 serves as the basic foundation of many of the top medical imaging models currently published, ranging from ophthalmology [5,6] and pathology [7] to dermatology [8].

In the past three weeks, a new AI model called ChatGPT garnered significant attention due to its ability to perform a diverse array of natural language tasks [9]. ChatGPT is a general Large Language Model (LLM) developed recently by OpenAI. While the previous class of AI models have primarily been Deep Learning (DL) models, which are designed to learn and recognize patterns in data, LLMs are a new type of AI algorithm trained to predict the likelihood of a given sequence of words based on the context of the words that come before it. Thus, if LLMs are trained on sufficiently large amounts of text data, they are capable of generating novel sequences of words never observed previously by the model, but that represent plausible sequences based on natural human language. ChatGPT is powered by GPT3.5, an LLM trained on the OpenAI 175B parameter foundation model and a large corpus of text data from the Internet via reinforcement and supervised learning methods. Anecdotal usage indicates that ChatGPT exhibits evidence of deductive reasoning and chain of thought, as well as long-term dependency skills.

In this study, we evaluate the performance of ChatGPT, a non-domain specific LLM, on its ability to perform clinical reasoning by testing its performance on questions from the United States Medical Licensing Examination (USMLE). The USMLE is a high-stakes, comprehensive three-step standardized testing program covering all topics in physicians’ fund of knowledge, spanning basic science, clinical reasoning, medical management, and bioethics. The difficulty and complexity of questions is highly standardized and regulated, making it an ideal input substrate for AI testing. The examination is well-established, showing remarkably stable raw scores and psychometric properties over the previous ten years [10]. The Step 1 exam is typically taken by medical students who have completed two years of didactic and problem-based learning and focuses on basic science, pharmacology, and pathophysiology; medical students often spend approximately 300–400 hours of dedicated study time in preparation for this exam [11]. The Step 2CK exam is usually taken by fourth-year medical students who have additionally completed 1.5 to 2 years of clinical rotations; it emphasizes clinical reasoning, medical management, and bioethics. The Step 3 exam is taken by physicians who generally have completed at least a 0.5 to 1 year of postgraduate medical education.

USMLE questions are textually and conceptually dense; text vignettes contain multimodal clinical data (i.e., history, physical examination, laboratory values, and study results) often used to generate ambiguous scenarios with closely-related differential diagnoses. Due to its linguistic and conceptual richness, we reasoned that the USMLE would serve as an excellent challenge for ChatGPT.

Our work aims to provide both qualitative and quantitative feedback on the performance of ChatGPT and assess its potential for use in healthcare.

## Methods

### Artificial Intelligence

ChatGPT (OpenAI; San Francisco, CA), is a large language model that uses self-attention mechanisms and a large amount of training data to generate natural language responses to text input in a conversational context. It is particularly effective at handling long-range dependencies and generating coherent and contextually appropriate responses. ChatGPT is a server-contained language model that is unable to browse or perform internet searches. Therefore, all responses are generated *in situ*, based on the abstract relationship between words (“tokens”) in the neural network. This contrasts to other chatbots or conversational systems that are permitted to access external sources of information (e.g. performing online searches or accessing databases) in order to provide directed responses to user queries.

### Input source

376 publicly-available test questions were from the June 2022 sample exam release, termed USMLE-2022, were obtained from the official USMLE website. Therefore, all inputs represented true out-of-training samples for the GPT3 model. This was further confirmed by randomly spot checking the inputs to ensure that none of the answers, explanations, or related content were indexed on Google prior to January 1, 2022, representing the last date accessible to the ChatGPT training dataset. All sample test questions were screened, and questions containing visual assets such as clinical images, medical photography, and graphs were removed. After filtering, 350 USMLE items (Step 1: 119, Step 2CK: 102, Step 3: 122) were advanced to encoding. Assuming a normal distribution of model performance, this affords 90% power at α = 0.05 to detect a 2.5% increase in accuracy against a baseline rate of 60 ± 20% (σ).

### Encoding

Questions were formatted into three variants and input into ChatGPT in the following sequence:

1. Open-ended (OE) prompting: Created by removing all answer choices, adding a variable lead-in interrogative phrase. This format simulates free input and a natural user query pattern. Examples include: “What would be the patient’s diagnosis based on the information provided?”; or “In your opinion, what is the reason for the patient’s pupillary asymmetry?”
2. Multiple choice single answer without forced justification (MC-NJ) prompting: Created by reproducing the original USMLE question verbatim. Examples include: “Which of the following best represent the most appropriate next step in management?”; or “The patient’s condition is mostly caused by which of the following pathogens?”
3. Multiple choice single answer with forced justification (MC-J) prompting: Created by adding a variable lead-in imperative or interrogative phrase mandating ChatGPT to provide a rationale for each answer choice. Examples include: “Which of the following is the most likely reason for the patient’s nocturnal symptoms? Explain your rationale for each choice”; or “The most appropriate pharmacotherapy for this patient most likely operates by which of the following mechanisms? Why are the other choices incorrect?”

Encoders employed deliberate variation in the lead-in prompts to avoid systematic errors introduced by rigid wording. To reduce memory retention bias, a new chat session was started in ChatGPT for each entry. Ordinary 2-way ANOVA of AI response accuracy were performed *post hoc* to evaluate for systematic covariation between encoders and question prompt type (**S3 Data**). Encoders were first considered as individuals (*n* = 8 inputters), and then subsequently as groups classified by level of medical expertise (*n =* 4 groups: physician, medical student, nurse, or nonmedical generalist).

### Adjudication

AI outputs were independently scored for Accuracy, Concordance, and Insight (ACI) by two physician adjudicators using the criteria enumerated in **S2 Data**. The physicians were blinded to each other. A subset of 20 USMLE questions were used for collective adjudicator training. Physicians were not blinded for this subset, but interrater cross-contamination was suppressed by forcing staggered review of output measures. For instance, Physician 1 adjudicated Accuracy while Physician 2 adjudicated Concordance. The roles were then rotated such that each adjudicator provided a complete ACI rating for the entire dataset. To minimize within-item anchoring bias, adjudicators scored Accuracy for all items, followed by Concordance for all items, followed by Insight for all items. If consensus was not achieved for all three domains, the item was referred to a final physician adjudicator. A total of 21 items (6.2% of the dataset) required arbitration by a third physician. Interrater agreement between physicians was evaluated by computing the Cohen kappa (κ) statistic for OE and MC questions (**S4 Data**).

**Fig 1:**
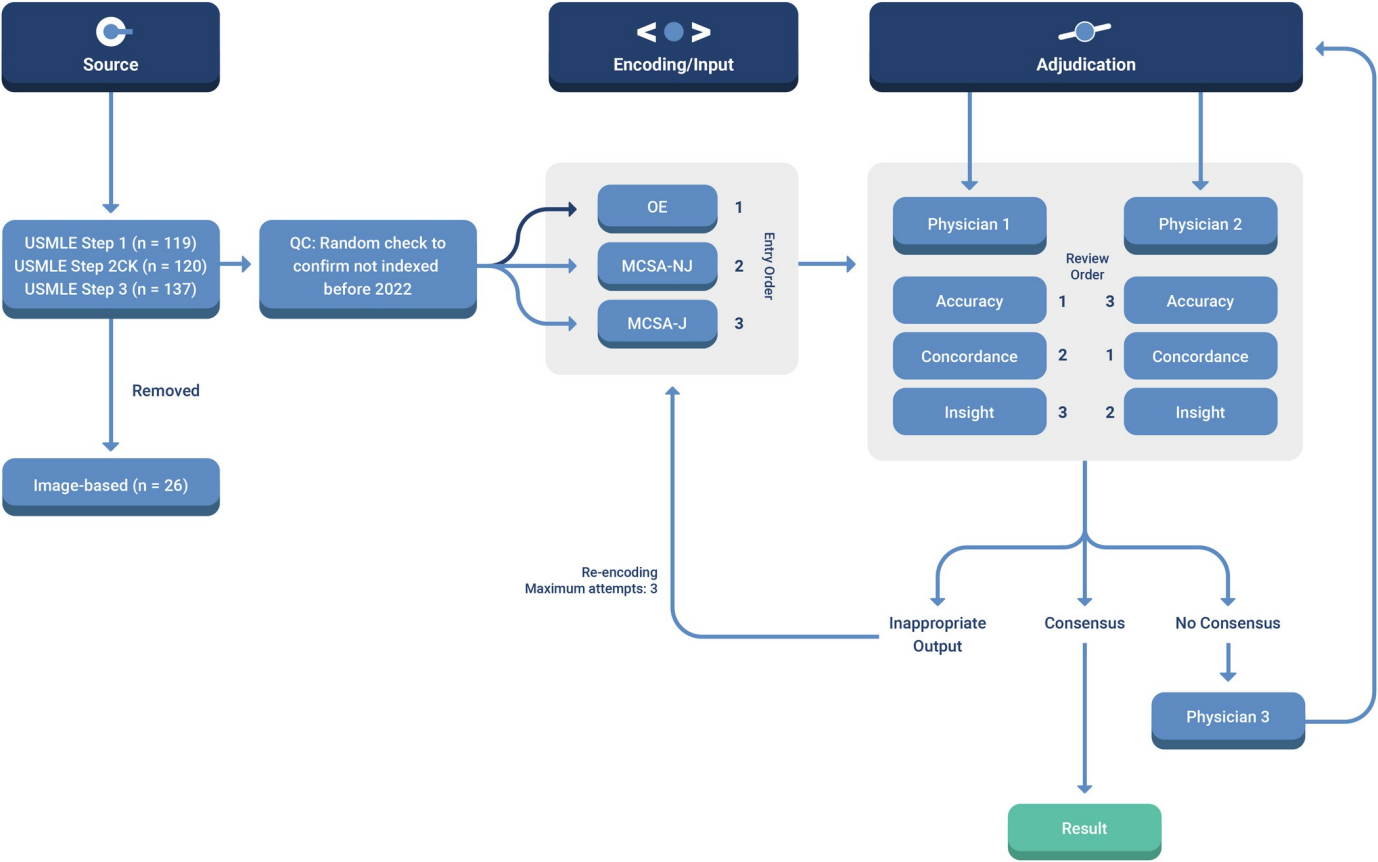
Schematic of workflow for sourcing, encoding, and adjudicating results. Abbreviations: **QC** = quality control; **MCSA-NJ** = multiple choice single answer without forced justification; **MCSA-J** = multiple choice single answer with forced justification; **OE** = open-ended question format.

A schematic overview of the study protocol is provided in **Fig 1**.

## Results

### ChatGPT yields moderate accuracy approaching passing performance on USMLE

**Fig 2:**
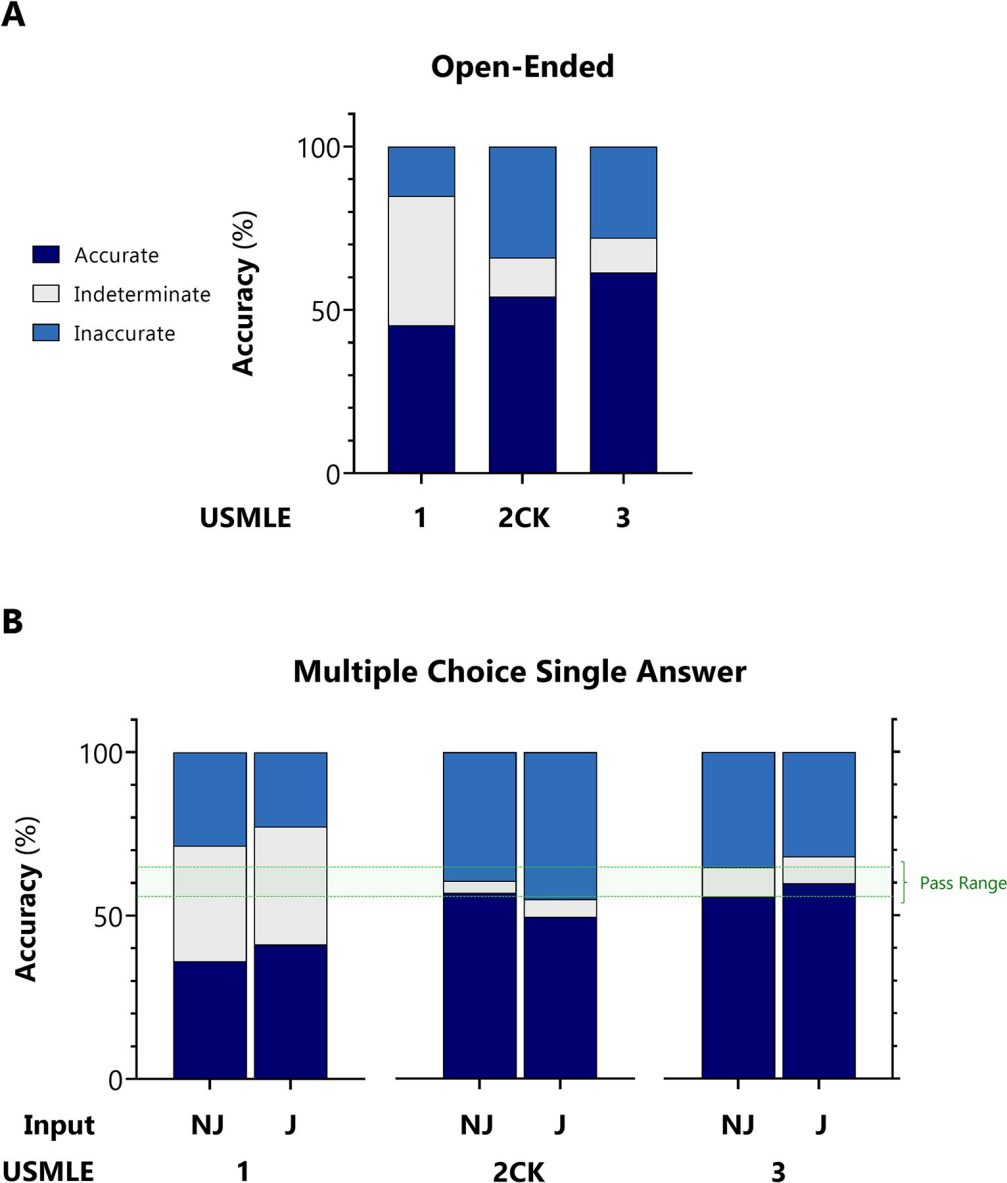
Accuracy of ChatGPT on USMLE. For USMLE Steps 1, 2CK, and 3, AI outputs were adjudicated to be accurate, inaccurate, or indeterminate based on the ACI scoring system provided in **S2 Data**. **A:** Accuracy distribution for inputs encoded as open-ended questions. **B:** Accuracy distribution for inputs encoded as multiple choice single answer without (MC-NJ) or with forced justification (MC-J).

Exam items were first encoded as open-ended questions with variable lead-in prompts. This input format simulates a free natural user query pattern. With indeterminate responses censored/included, ChatGPT accuracy for USMLE Steps 1, 2CK, and 3 was 75.0%/45.4%, 61.5%/54.1%, and 68.8%/61.5%, respectively (**Fig 2A**).

Next, exam items were encoded as multiple choice single answer questions with no forced justification (MC-NJ). This input is the verbatim question format presented to test-takers. With indeterminate responses censored/included, ChatGPT accuracy for USMLE Steps 1, 2CK, and 3 was 55.8%/36.1%, 59.1%/56.9%, and 61.3%/55.7%, respectively.

Finally, items were encoded as multiple choice single answer questions with forced justification of positive and negative selections (MC-J). This input format simulates insight-seeking user behavior. With indeterminate responses censored/included, ChatGPT accuracy was 64.5%/ 41.2%, 52.4%/49.5%, and 65.2%/59.8%, respectively (**Fig 2B**).

At the encoding stage, there were no statistically significant interactions between encoders and question prompt type, regardless of whether encoders were analyzed as individuals or when grouped by level of medical expertise (**S3 Data**). As expected, inter-individual variation dominated over inter-group variation, but the overall contribution was insignificant relative to residual error. At the adjudication stage, physician agreement was substantial for OE prompts (κ range from 0.74 to 0.81) and nearly perfect for MC prompts (κ >0.9) (**S4 Data**).

### ChatGPT demonstrates high internal concordance

**Fig 3:**
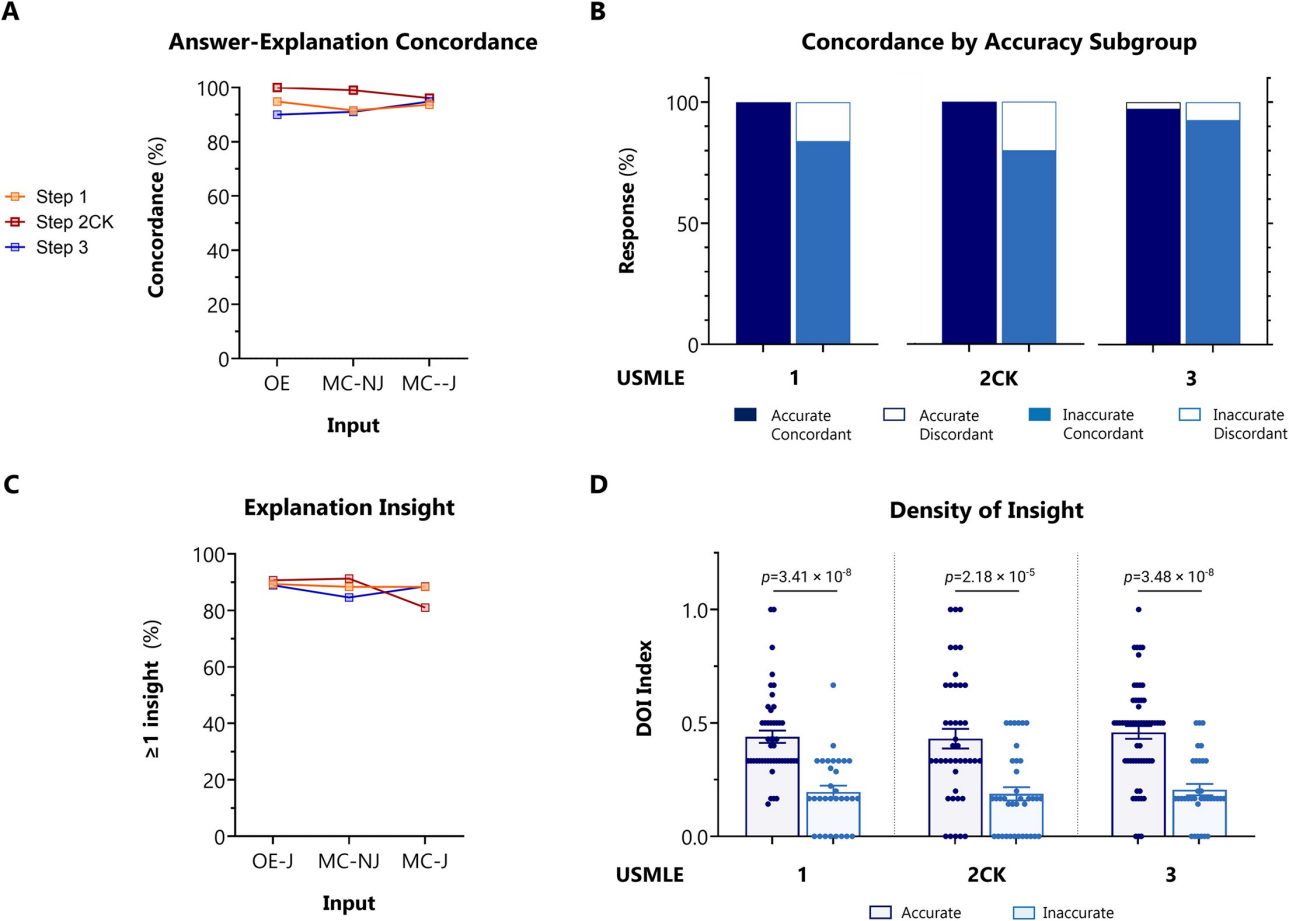
Concordance and insight of ChatGPT on USMLE. For USMLE Steps 1, 2CK, and 3, AI outputs were adjudicated on concordance and density of insight (DOI) based on the ACI scoring system provided in **S2 Data**. **A:** Overall concordance across all exam types and question encoding formats. **B:** Concordance rates stratified between accurate *vs* inaccurate outputs, across all exam types and question encoding formats. *p* <0.001 for accurate *vs* inaccurate outputs by Fisher exact test. **C:** Overall insight prevalence, defined as proportion of outputs with ≥1 insight, across all exams for questions encoded in MC-J format. **D:** DOI stratified between accurate *vs* inaccurate outputs, across all exam types for questions encoded in MC-J format. Horizontal line indicates the mean. *p*-value determined by parametric 2-way ANOVA testing with Benjamini-Krieger-Yekutieli (BKY) *post hoc* to control for false discovery rate.

Concordance was independently adjudicated by two physician reviewers by inspection of the explanation content. Overall, ChatGPT outputted answers and explanations with 94.6% concordance across all questions. High global concordance was sustained across all exam levels, and across OE, MC-NJ, and MC-J question input formats (**Fig 3A**).

Next, we analyzed the contingency between accuracy and concordance in MC-J responses. ChatGPT was forced to justify its answer choice preference, and to defend its rejection of alternative choices. Concordance amongst accurate responses was nearly perfect, and significantly greater than amongst inaccurate responses (99.1% vs. 85.1%, *p*<0.001) (**Fig 3B**).

These data indicate that ChatGPT exhibits very high answer-explanation concordance, likely reflecting high internal consistency in its probabilistic language model.

### Explanations generated by ChatGPT contain nonobvious insights

Having established the accuracy and concordance of ChatGPT, we next examined its potential to augment human learning in the domain of medical education. AI-generated explanations were independently adjudicated by 2 physician reviewers. Explanation content was examined for significant insights, defined as instances that met the criteria (see **S2 Data**) of *novelty*, *nonobviousness*, and *validity*. The perspective of the target test audience was adopted by the adjudicator, as a second-year medical student for Step 1, fourth-year medical student for Step 2CK, and post-graduate year 1 resident for Step 3.

We first examined the frequency (prevalence) of insight. Overall, ChatGPT produced at least one significant insight in 88.9% of all responses. Insight frequency was generally consistent between exam type and question input format (**Fig 3C**). In Step 2CK however, insight decreased by 10.3% (*n* = 11 items) between MC-NJ and MC-J formulations, paralleling the decrement in accuracy (**Fig 1B**). Review of this subset of questions did not reveal a discernible pattern for the paradoxical decrease (see specifically annotated items [*] in **S1 Data**).

Next, we quantified the density of insight (DOI) contained within AI-generated explanations. A density index was defined by normalizing the number of unique insights against the number of possible answer choices. This analysis was performed on MC-J entries only. High quality outputs were generally characterized by DOI >0.6 (i.e. unique, novel, nonobvious, and valid insights provided for >3 out of 5 choices); low quality outputs were generally characterized by DOI ≤0.2. The upper limit on DOI is only bounded by the maximum length of text output. Across all exam types, we observed that mean DOI was significantly higher in questions items answered accurately versus inaccurately (0.458 versus 0.199, *p* <0.0001) (**Fig 3D**).

The high frequency and moderate density of insights indicate that it may be possible for a target learner (e.g., such as a second-year medical student preparing for Step 1) to gain new or remedial knowledge from the ChatGPT AI output, particularly if answering incorrectly.

## Discussion

In this study, we provide new and surprising evidence that ChatGPT is able to perform several intricate tasks relevant to handling complex medical and clinical information. To assess ChatGPT’s capabilities against biomedical and clinical questions of *standardized* complexity and difficulty, we tested its performance characteristics on the United States Medical Licensing Examination (USMLE).

Our findings can be organized into two major themes: (1) the rising accuracy of ChatGPT, which approaches or exceeds the passing threshold for USMLE; and (2) the potential for this AI to generate novel insights that can assist human learners in a medical education setting.

### The rising accuracy of ChatGPT

The most recent iteration of the GPT LLM (GPT3) achieved 46% accuracy with zero prompting [12], which marginally improved to 50% with further model training and extensive prompt tuning. Previous models, merely months prior, performed at 36.7% [13]. In this present study, ChatGPT performed at >50% accuracy across all examinations, exceeding 60% in some analyses. The USMLE pass threshold, while varying by year, is approximately 60%. Therefore, ChatGPT now approaches the passing range. Being the first experiment to reach this benchmark, we believe this is a surprising and impressive result. Moreover, we provided no prompting or training to the AI, minimized grounding bias by expunging the AI session prior to inputting each question variant, and avoided chain-of-thought biasing by requesting forced justification only as the final input. Further model interaction and prompt tuning could often produce more accurate results. Given this trajectory, it is likely that AI performance will continue to improve as LLM models continue to mature.

Paradoxically, ChatGPT outperformed PubMedGPT [14] (accuracy 50.3%), a counterpart LLM with similar neural structure, but trained exclusively on biomedical domain literature. We speculate that domain-specific training may have created greater ambivalence in the PubMedGPT model, as it absorbs real-world text from ongoing academic discourse that tends to be inconclusive, contradictory, or highly conservative or noncommittal in its language. A foundation LLM trained on general content, such as ChatGPT, may therefore have an advantage because it is also exposed to broader clinical content, such as patient-facing disease primers and provider-facing drug package inserts, that are more definitive and congruent.

An additional explanation for the observed difference in performance may be the disparate AI testing datasets. Our present study tested ChatGPT against contemporary USMLE examinations (publicly available no earlier than 2022, 5 answer choices per question), whereas previous reports tested language models against the MedQA-USMLE dataset [13] (publicly available 2009–2020, 4 answer choices per question). Although we did not perform a direct comparison against MedQA-UMSLE, our approach nonetheless has several advantages. It is guaranteed that none of our inputs were previously seen by GPT3, whereas many of the inputs from MedQA-USMLE would have likely been ingested during model pretraining. Considering that medical knowledge proliferates at a faster-than-exponential rate [15] and previous evidence-based practice is frequently debunked [16,17], some concepts tested by MedQA-USMLE are already antiquated and not representative of present-day examination content. Finally, the higher accuracy of ChatGPT on USMLE-2022 despite a greater number of answer choices (5 versus 4) may indicate even more impressive performance of this model relative to other domain-specific language models such as PubMedGPT and BioBERT.

Consistent with the mechanism of generative language models, we observed that the accuracy of ChatGPT was strongly mediated by concordance and insight. High accuracy outputs were characterized by high concordance and high density of insight. Poorer accuracy was characterized by lower concordance and a poverty of insight. Therefore, inaccurate responses were driven primarily by missing information, leading to diminished insight and indecision in the AI, rather than overcommitment to the incorrect answer choice. These findings indicate that model performance could be significantly improved by merging foundation models, such as ChatGPT, with a domain-specific LLM or other model trained on a voluminous and highly validated medical knowledge resources, such as UpToDate, or other ACGME-accredited content.

Interestingly, the accuracy of ChatGPT tended to be lowest for Step 1, followed by Step 2CK, followed by Step 3. This mirrors both the subjective difficulty and objective performance for real-world test takers on Step 1, which is collectively regarded as the most difficult exam of the series. The low accuracy on Step 1 could be explained by an undertrained model on the input side (e.g. underrepresentation of basic science content on the general information space) and/or the human side (e.g. insufficient or invalid human judgment at initial reinforcement stages). This result exposes a key vulnerability in pre-trained LLMs, such as ChatGPT: AI ability becomes yoked to human ability. ChatGPT’s performance on Step 1 is poorer precisely because human users perceive its subject matter (e.g., pathophysiology) as more difficult or opaque.

### The potential for AI-assisted human learning in medical education

We also examined the ability of ChatGPT to assist the human learning process of its target audience (e.g., a second year medical student preparing for USMLE Step 1). As a proxy for the metric of helpfulness, we assessed the concordance and insight offered by the AI explanation outputs. ChatGPT responses were highly concordant, such that a human learner could easily follow the internal language, logic, and directionality of relationships contained within the explanation text (e.g., adrenal *hyper*cortisolism ⥬ *increased* bone osteoclast activity ⥬ *increased* calcium resorption ⥬ *decreased* bone mineral density ⥬ *increased* fracture risk). High internal concordance and low self-contradiction is a proxy of sound clinical reasoning and an important metric of explanation quality. It is reassuring that the directionality of relationships is preserved by the language processing model, where each verbal object is individually lemmatized.

AI-generated responses also offered significant insight, role-modeling a deductive reasoning process valuable to human learners. At least one significant insight was present in approximately 90% of outputs. ChatGPT therefore possesses the partial ability to teach medicine by surfacing novel and nonobvious concepts that may not be in learners’ sphere of awareness. This qualitative gain provides a basis for future real-world studies on the efficacy of generative AI to augment the human medical education process. For example, longitudinal exam performance can be studied in a quasi-controlled in AI-assisted and unassisted learners. Unit economic analysis may clarify the cost-effectiveness of incremental student performance gain in comparison to existing tools such as virtual tutors and study aids.

Medical education, licensing examinations, and test preparation services form a large industrial complex eclipsing a nine-figure market size annually. While its relevance remains debated, standardized testing has emerged as an important end-target of medical learning. In parallel, of the didactic techniques, a socratic teaching style is favored by medical students [18]. The rate-limiting step for fresh content generation is the human cognitive effort required to craft realistic clinical vignettes that probe “high-yield” concepts in a subtle way, engage critical thinking, and offer pearls of knowledge even if answered incorrectly. Demand for new examination content continues to increase. Future studies may investigate the ability of generative language AI to offload this human effort by assisting in the question-explanation writing process or, in some cases, writing entire items autonomously.

Finally, the advent of AI in medical education demands an open science research infrastructure to standardize experimental methods, readouts, and benchmarks to describe and quantify human-AI interactions. Multiple dimensions must be covered, including user experience, learning environment, hybridization with other teaching modes, and effect on cognitive bias. In this report, we provide an initial basic protocol for adjudicating AI-generated responses along axes of accuracy, concordance, and insight.

Our study has several important limitations. The relatively small input size restricted the depth and range of analyses. For example, stratifying the output of ChatGPT by subject taxonomy (e.g., pharmacology, bioethics) or competency type (e.g., differential diagnosis, management) may be of great interest to medical educators, and could reveal heterogeneities in performance across language processing for different clinical reasoning tasks. Similarly, a more robust AI failure mode analysis (e.g., language parsing error) may lend insight into the etiology of inaccuracy and discordance. In addition to being laborious, human adjudication is error-prone and subject to greater variability and bias. Future studies will undoubtedly apply unbiased approaches, using quantitative natural language processing and text mining tools such as word network analysis. In addition to increasing validity and accelerating throughput by several orders of magnitude, these methods are likely to better characterize the depth, coherence, and learning value of AI output. Finally, to truly assess the utility of generative language AI for medical education, ChatGPT and related applications must be studied in both controlled and real-world learning scenarios with students across the engagement and knowledge spectrum.

Beyond their utility for medical education, AIs are now positioned to soon become ubiquitous in clinical practice, with diverse applications across all healthcare sectors. Investigation of AI has now entered into the era of randomized controlled trials [19]. Additionally, a profusion of pragmatic and observational studies supports a versatile role of AI in virtually all medical disciplines and specialties by improving risk assessment [20,21], data reduction, clinical decision support [22,23], operational efficiency, and patient communication [24,25].

Inspired by the remarkable performance of ChatGPT on the USMLE, clinicians within AnsibleHealth, a virtual chronic pulmonary disease clinic, have begun to experiment with ChatGPT as part of their workflows. Inputting queries in a secure and de-identified manner, our clinicians request ChatGPT to assist with traditionally onerous writing tasks such as composing appeal letters to payors, simplifying radiology reports (and other jargon-dense records) to facilitate patient comprehension, and even to brainstorm and kindle insight when faced with nebulous and diagnostically challenging cases. We believe that LLMs such as ChatGPT are reaching a maturity level that will soon impact clinical medicine at large, enhancing the delivery of individualized, compassionate, and scalable healthcare.

## Supporting information

S1 Data Raw data files containing unprocessed question inputs and ChatGPT outputs. (PDF)

S2 Data Adjudication criteria for accuracy, concordance, and insight. (PDF)

S3 Data ANOVA for systematic encoder effects. (PDF)

S4 Data Kappa statistic for interrater agreement between adjudicating physicians. (PDF)
