# Supplementary material for: Performance of ChatGPT on USMLE: Potential for AI-assisted medical education using large language models
Source: PLOS Digit Health. 2023 Feb 9;2(2):e0000198. doi: 10.1371/journal.pdig.0000198 (PMC9931230; doi:10.1371/journal.pdig.0000198)
Supplement: S1 Data — (PDF) [file pdig.0000198.s001.pdf]

The raw data file can be accessed at the following URLs	
**Step 1**	https://tinyurl.com/544jeebc
**Step 2CK**	https://tinyurl.com/yjjczmwd
**Step 3**	https://tinyurl.com/es9m8dfa

Suppporting Information 1
