# Supplementary material for: Performance of ChatGPT on USMLE: Potential for AI-assisted medical education using large language models
Source: PLOS Digit Health. 2023 Feb 9;2(2):e0000198. doi: 10.1371/journal.pdig.0000198 (PMC9931230; doi:10.1371/journal.pdig.0000198)
Supplement: S2 Data — (PDF) [file pdig.0000198.s002.pdf]

<table>
<tr><th colspan="2">Adjudication Criteria: A-C-I Scoring System</th></tr>
<tr><td rowspan="4">Accuracy</td><td>MC-NJ and MC-J</td></tr>
<tr><td>Accurate: Final answer matches the NBME key
Inaccurate: Incorrect answer choice is selected
Indeterminate: At least one of the following occurs:
AI output is not an answer choice
AI returns a
AI determines that not enough information is available</td></tr>
<tr><td>OE</td></tr>
<tr><td>Accurate: All of the following are true:
AI response identifies the central concept being tested
AI response is specific
AI response is clinically sound and meets the reasonable physician standard
Inaccurate: Response targets an unrelated concept or is not clinically sound
Indeterminate: Any other response, including generic information</td></tr>
<tr><td rowspan="4">Concordance</td><td>MC-J</td></tr>
<tr><td>Concordant: Explanation affirms the answer and negates <u>all</u> remaining choices
Discordant: <u>Any</u> part of the explanation contradicts itself</td></tr>
<tr><td>MC-NJ and OE</td></tr>
<tr><td>Concordant: Explanation affirms the answer
Discordant: <u>Any</u> part of the explanation contradicts itself</td></tr>
<tr><td rowspan="2">Insight</td><td>Insight: An instance of text in the explanation that is:
Nondefinitional: Does not simply define a term in the input question
Unique: A single insight may be used to eliminate several answer choices
Nonobvious: Requires deduction or knowledge external to the question input
Valid: In clinically or numerically accurate; preserves directionality
Density of Insight (DOI): Number of insights / (Number of answer choices + 1)</td></tr>
<tr><td>Insightful: DOI ≥1
Non-insightful: DOI = 0</td></tr>
</table>

Supporting Information 2
