# Supplementary material for: Performance of ChatGPT on USMLE: Potential for AI-assisted medical education using large language models
Source: PLOS Digit Health. 2023 Feb 9;2(2):e0000198. doi: 10.1371/journal.pdig.0000198 (PMC9931230; doi:10.1371/journal.pdig.0000198)
Supplement: S3 Data — (PDF) [file pdig.0000198.s003.pdf]

A

Encoder (Individual)	Accuracy (%) OE	MC-NJ	MC-J
A	54.84	59.09	60.71
B	52.73	55.36	54.55
C	55.88	56.72	52.86
D	54.29	54.29	57.14
E	62.5	59.38	76.67
F	48.57	69.7	67.74
G	61.11	61.11	57.14
H	63.41	52.5	48.65

Source of Variation	% Total variation	*p*
Encoder (Individual)	38.39	0.3128
Prompt Type	3.232	0.6858

Ordinary 2-way ANOVA with α = 0.05

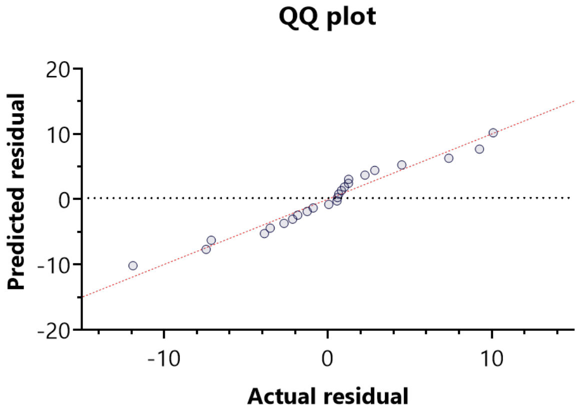

B

Encoder (Expertise)	Accuracy (%) OE	MC-NJ	MC-J
Physician	54.84	59.09	60.71
Medical Student	52.23	63.21	60.30
Nurse	57.70	57.70	57.14
Generalist	59.55	55.75	59.96

Source of Variation	% Total variation	*p*
Encoder (Expertise)	2.071	0.9793
Prompt Type	28.31	0.3593

Ordinary 2-way ANOVA with α = 0.05

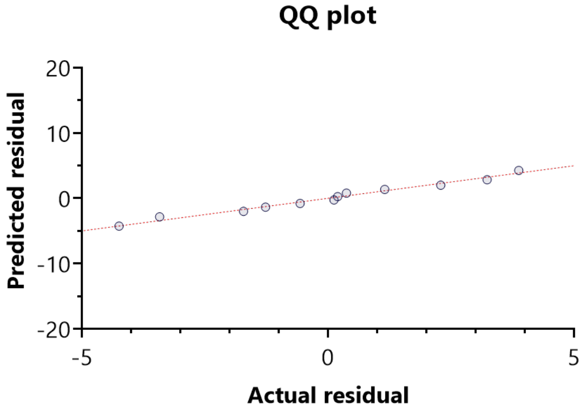

Supporting Information 3
