# Supplementary material for: Performance of ChatGPT on USMLE: Potential for AI-assisted medical education using large language models
Source: PLOS Digit Health. 2023 Feb 9;2(2):e0000198. doi: 10.1371/journal.pdig.0000198 (PMC9931230; doi:10.1371/journal.pdig.0000198)
Supplement: S4 Data — (PDF) [file pdig.0000198.s004.pdf]

	OE		MC-NJ + MC-J	
	Cohen κ	*n*	Cohen κ	*n*
**Step 1**	**0.782**	119	**0.986**	238
**Step 2CK**	**0.747**	109	**0.978**	218
**Step 3**	**0.813**	122	**0.947**	244

Supporting Information 4
